# DNA Topoisomerase III Localizes to Centromeres and Affects Centromeric CENP-A Levels in Fission Yeast

**DOI:** 10.1371/journal.pgen.1003371

**Published:** 2013-03-14

**Authors:** Ulrika Norman-Axelsson, Mickaël Durand-Dubief, Punit Prasad, Karl Ekwall

**Affiliations:** Center for Biosciences, Department of Biosciences and Nutrition, Karolinska Institutet, Huddinge, Sweden; University of Virginia, United States of America

## Abstract

Centromeres are specialized chromatin regions marked by the presence of nucleosomes containing the centromere-specific histone H3 variant CENP-A, which is essential for chromosome segregation. Assembly and disassembly of nucleosomes is intimately linked to DNA topology, and DNA topoisomerases have previously been implicated in the dynamics of canonical H3 nucleosomes. Here we show that *Schizosaccharomyces pombe* Top3 and its partner Rqh1 are involved in controlling the levels of CENP-A^Cnp1^ at centromeres. Both *top3* and *rqh1* mutants display defects in chromosome segregation. Using chromatin immunoprecipitation and tiling microarrays, we show that Top3, unlike Top1 and Top2, is highly enriched at centromeric central domains, demonstrating that Top3 is the major topoisomerase in this region. Moreover, centromeric Top3 occupancy positively correlates with CENP-A^Cnp1^ occupancy. Intriguingly, both *top3* and *rqh1* mutants display increased relative enrichment of CENP-A^Cnp1^ at centromeric central domains. Thus, Top3 and Rqh1 normally limit the levels of CENP-A^Cnp1^ in this region. This new role is independent of the established function of Top3 and Rqh1 in homologous recombination downstream of Rad51. Therefore, we hypothesize that the Top3-Rqh1 complex has an important role in controlling centromere DNA topology, which in turn affects the dynamics of CENP-A^Cnp1^ nucleosomes.

## Author Summary

Centromeres are unique regions on eukaryotic chromosomes that are essential for chromosome segregation at mitosis and meiosis. Centromere identity and function depends on the presence of specialized chromatin with nucleosomes containing the centromere-specific histone H3 variant CENP-A. Assembly and disassembly of nucleosomes have previously been shown to involve a family of enzymes known as DNA topoisomerases. We show that centromeres are unique in that they are associated with high levels of Top3, but low levels of Top1 and Top2, suggesting that Top3 is particularly important for centromeric DNA topology. Impaired function of Top3 or its partner Rqh1 results in chromosome segregation defects and increased levels of CENP-A^Cnp1^ at centromeres. This role in limiting the levels of CENP-A^Cnp1^ at centromeres is independent of the established role for the Top3-Rqh1 complex in homologous recombination. Therefore, we hypothesize that the Top3-Rqh1 complex exerts this effect by regulating centromere DNA topology, which in turn affects CENP-A^Cnp1^ nucleosome dynamics. Specific removal of negative supercoiling by Top3 could directly have a negative effect on assembly of CENP-A^Cnp1^ nucleosomes with left-handed negative wrapping of DNA and/or act indirectly by inhibiting transcription-coupled CENP-A^Cnp1^ assembly. Alternatively, Top3 may be a factor that promotes formation of CENP-A^Cnp1^ hemisomes with right-handed wrapping of DNA over conventional octamers. This suggests a new role for the Top3-Rqh1 complex at centromeres and may contribute to the understanding of the structural and functional specification of centromeres.

## Introduction

Centromeres are unique regions of eukaryotic chromosomes that are essential for chromosome segregation at mitosis and meiosis. The specialized centromeric chromatin directs assembly of kinetochores, which serve as points of attachment for the spindle apparatus. In all eukaryotes, centromeric chromatin is marked by the presence of nucleosomes containing the histone H3 variant Centromere Protein-A (CENP-A), which is a key determinant for centromere identity and essential for centromere function. The specific incorporation and maintenance of CENP-A at centromeres is an epigenetic phenomenon that is not yet fully understood, and new factors involved in CENP-A dynamics are continuing to be discovered. During DNA replication in S-phase pre-existing CENP-A is equally partitioned to sister centromeres and after chromosome segregation newly synthesized CENP-A is incorporated specifically at pre-existing centromeres, possibly involving a feed-forward mechanism between pre-existing CENP-A chromatin and CENP-A assembly factors [1]–[2]. In agreement, it was recently shown that the constitutive centromere-associated network (CCAN) component Centromere Protein C (CENP-C) is required for recruitment of the Mis18 complex [3]. The Mis18 complex is in turn necessary for localization of CENP-A to centromeres and is hypothesized to have a role in centromere priming [4]–[7]. Furthermore, Mis18 is required for centromere targeting of the CENP-A-specific chaperone Holliday Junction Recognition Protein (HJURP), which is both necessary and sufficient for stable recruitment of CENP-A [6], [8]–[9]. It binds specifically to pre-nucleosomal CENP-A and histone H4, and has been shown to facilitate assembly of nucleosomes containing CENP-A *in vitro* [8]–[14]. The Mis18 complex and/or the CENP-A pre-nucleosomal complex also associates with the chaperone RbAp48, which can mediate assembly of nucleosomes containing CENP-A *in vitro* [4]–[5], [10]–[11], [15].

Despite continuous advances in the identification of pathways and factors controlling CENP-A nucleosome assembly, the molecular architecture of CENP-A nucleosomes remains unclear. CENP-A, H4, H2A and H2B can be assembled into conventional octameric nucleosomes with left-handed negative wrapping of DNA *in vitro* [13], [16]–[19]. However, they can also be assembled into tetrameric hemisomes containing only one copy of each histone with right-handed positive wrapping of DNA [20]. Different studies aiming at determining the composition of CENP-A nucleosomes *in vivo* have found contradicting evidence for both octamers and tetramers [17], [20]–[24]. Interestingly, recent studies imply that there may be cell cycle-dependent transitions in the structure of CENP-A nucleosomes *in vivo* [25]–[26]. This would reconcile the contradictions between different studies on CENP-A nucleosome structure. A few other models for the structure of CENP-A nucleosomes have also been proposed, but are associated with less experimental evidence.

DNA topoisomerases catalyze changes in DNA topology by cutting, shuffling and re-ligating DNA strands. Nucleosome dynamics are intimately linked to DNA topology [27]. Since DNA is wrapped around the histone core of nucleosomes in a left-handed negative direction, negative supercoiling of DNA favors nucleosome assembly while positive supercoiling of DNA favors nucleosome disassembly. In agreement, DNA topoisomerases have been implicated in the dynamics of canonical H3 nucleosomes *in vitro* and *in vivo* [28]–[31]. Eukaryotic topoisomerase III is a type 1A topoisomerase capable of relaxing negatively supercoiled DNA [32]–[33]. Topoisomerase III displays evolutionarily conserved genetic and physical interactions with RecQ helicases and RecQ-mediated genome instability (Rmi) proteins [34]–[39]. RecQ helicases and Rmi1 stimulate relaxation of negative supercoiling by topoisomerase III and together they also have the ability to fully de-catenate and catenate DNA molecules as well as to ‘dissolve’ double Holiday Junctions [40]–[43]. The single RTR complex in *Schizosaccharomyces pombe* consists of Rqh1, Top3 and Rmi1. These proteins are critical for genome stability and have so far been implicated in homologous recombination (HR) and DNA damage checkpoint activation *in vivo* [35], [44]–[50]. Both *top3* and *rmi1* deletion mutants stop dividing after just a few generations with severe defects in nuclear morphology and chromosome segregation [37], [51]–[52]. Thermo-sensitive *top3* mutants display growth defects, sensitivity to DNA damaging agents, illegitimate recombination, altered nuclear morphology, and defects in chromosome segregation at restrictive temperatures [46], [53]. The lethality of *top3* and *rmi1* deletion mutants can be rescued by mutations in *rqh1*, likely because Rqh1 creates intermediate structures in HR that without Top3 remain unresolved and prevent chromosome segregation [51]–[52]. Mutations in *rqh1* result in similar but less severe phenotypes compared to *top3* and *rmi1* mutations [49], [54]–[55].

In this report, we investigated the genome-wide localization of *S. pombe* Top3 and discovered that it is preferentially found at intergenic regions (IGRs), sub-telomeres and centromeres. Top3 occupancy at IGRs is similar to that of Top1 and Top2. On the other hand, high relative enrichment of Top3 at centromeric central domains is unique, and is positively correlated with CENP-A^Cnp1^ occupancy. Both *top3* and *rqh1* mutants display defects in chromosome segregation and increased relative enrichment of CENP-A^Cnp1^ at centromeric central domains. Thus, the Top3-Rqh1 complex normally limits the levels of CENP-A^Cnp1^ in this region. Altered levels of CENP-A^Cnp1^ are accompanied by changes in the levels of the CENP-A^Cnp1^-specific chaperone HJURP^Scm3^ and are independent of HR downstream of Rad51. We therefore suggest that the Top3-Rqh1 complex has an important role in controlling centromere DNA topology and thereby the dynamics CENP-A^Cnp1^ nucleosomes. Specific removal of negative supercoiling by Top3 should inhibit assembly of and destabilize octameric CENP-A^Cnp1^ nucleosomes with left-handed negative wrapping of DNA. In addition, removal of negative supercoiling may limit centromeric transcription, which is hypothesized to promote CENP-A^Cnp1^ nucleosome assembly. In this model, impaired Top3 activity would facilitate assembly of CENP-A^Cnp1^ nucleosomes. Alternatively, the activity of Top3 may create a unique topological state at centromeres that specifically favors formation of tetrameric CENP-A^Cnp1^ hemisomes with right-handed positive wrapping of DNA over octameric CENP-A^Cnp1^ nucleosomes. In this model, impaired Top3 activity would result in a shift from formation of CENP-A^Cnp1^-containing hemisomes towards octameres at centromeres.

## Results

### The *top3-105* mutant displays impaired growth and defects in chromosome segregation

**Figure 1:**
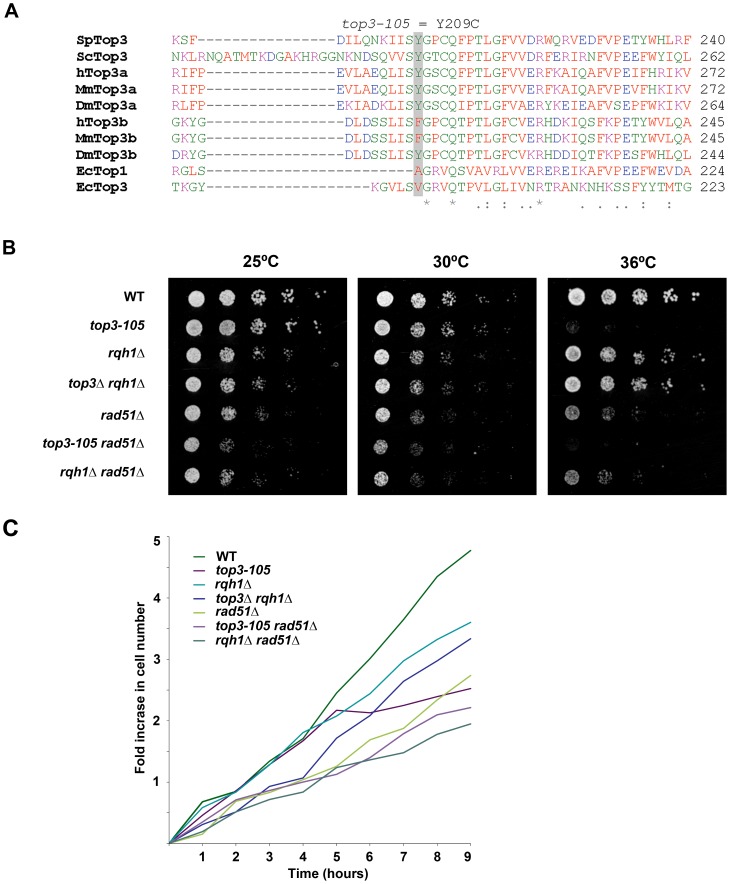
The *top3-105* mutant carries an Y209C amino acid substitution and displays impaired growth at 36°C. (A) Alignment of Top3 amino acid sequences from different species. The position corresponding to Y209 of *S. pombe* Top3 is highlighted in grey. The amino acids are colored according to their physiochemical properties. An asterisk (*) indicates a fully conserved residue, a colon (:) indicates conservation between groups of strongly similar properties, and a period (.) indicates conservation between groups of weakly similar properties between all species. (B) Spotting of the indicated strains in 5-fold serial dilutions on plates incubated at 25°C, 30°C and 36°C. (C) Growth kinetics of the indicated strains in liquid media after a shift from 25°C to 36°C at time zero.

In this study we used the previously isolated but uncharacterized thermo-sensitive *top3-105* mutant [56]. We sequenced the *top3* open reading frame and identified a A762G base pair substitution that results in a Tyr209Cys amino acid change (Figure 1A). This residue is found in a region involved in conformation changes upon DNA binding and is conserved in budding yeast Top3 as well as in metazoan Top3α [57]. Growth of the *top3-105* mutant is similar to wild type at 25°C and 30°C, but severely impaired at the restrictive temperature of 36°C (Figure 1B). When cultures grown at 25°C are shifted to 36°C, the *top3-105* mutant initially proliferates with kinetics similar to wild type, but after approximately two generations proliferation is severely slowed, with essentially no further increase in cell numbers (Figure 1C). Deletion of *rqh1* rescues the lethality of a *top3* deletion [51]–[52]. Growth of the *top3Δ rqh1Δ* double mutant is similar to the *rqh1Δ* mutant and somewhat slower compared to wild type at all temperatures (Figure 1B and 1C). The lethality of a *top3* deletion can also be suppressed by deleting *rad51*, which is upstream of Top3 and Rqh1 in the ‘dissolution’ pathway in HR [35]. The *rad51Δ* single mutant displays slow growth, which is further enhanced at 36°C, but not to the same extent as for the *top3-105* mutant (Figure 1B and 1C) [58]. The severe growth defect of the *top3-105* mutant is not suppressed by deletion of *rad51* as the *top3-105 rad51Δ* double mutant displays similarly impaired growth as the *top3-105* mutant at 36°C. Moreover, the *top3-105 rad51Δ* double mutant displays a small synthetic growth defect at 25°C and 30°C. Growth of the *rqh1Δ rad51Δ* double mutant is largely similar to the *rad51Δ* single mutant at all temperatures [59].

**Figure 2:**
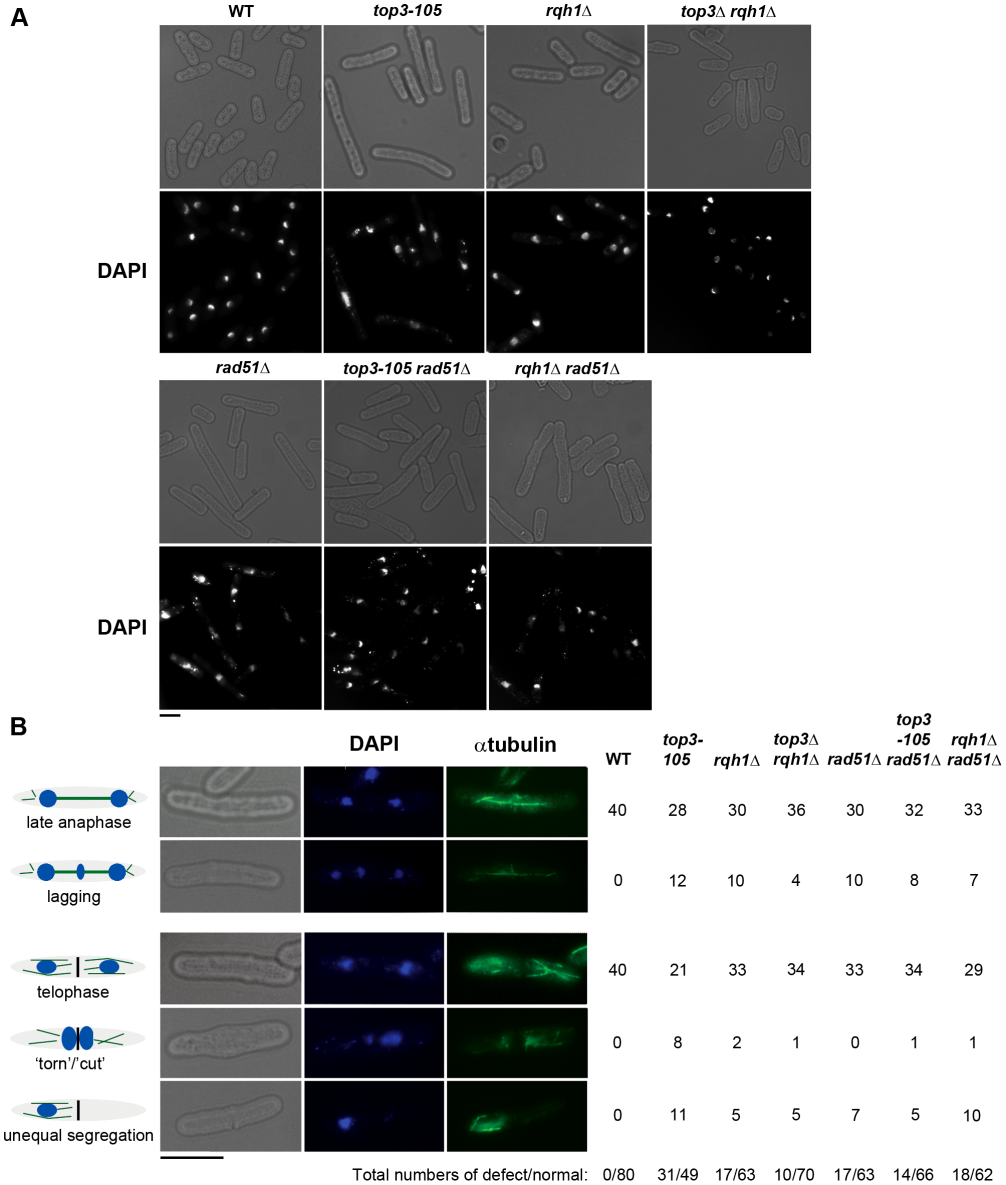
Top3 and Rqh1 are required for chromosome segregation. (A) Light and fluorescence microscopy images of the indicated strains with DAPI staining of the DNA after 8 hours at 36°C. (B) Light and fluorescence microscopy images of wild type and *top3-105* mutant cells after 8 hours at 36°C. The table shows the numbers of cells displaying normal and defective chromosome segregation among 40 late anaphase cells (with a mitotic spindle and two separate foci of DNA) or 40 telophase cells (with a septum) for the indicated strains after 8 hours at 36°C. The scale bars represents 6.65 µM.

Subsequently, we looked at nuclear morphology in the mutants using 4′.6-diamidino-2-phenylindole (DAPI) to stain DNA and anti-tubulin immunofluorescence to stain the mitotic spindle, respectively. After 8 hours at 36°C, there is a large increase in the fraction of abnormally long cells (>15 µm) for the *top3-105* mutant (28%, n = 500) compared to wild type (0.4%, n = 500), which is indicative of cell cycle delay (Figure 2A). Both the *rqh1Δ* and *top3Δ rqh1Δ* mutants display less pronounced increases in the fractions of unusually elongated cells (14% and 17%, respectively, n = 500). The *rad51Δ*, *rad51Δ top3-105* and *rad51Δ rqh1Δ* mutants all display equally large fractions of elongated cells (53%, 52% and 53%, respectively, n = 500) [58]. Furthermore, the *top3-105* mutant display various nuclear and mitotic defects, including amorphous and fragmented nuclei, unequal segregation of DNA, lagging chromosomes and a ‘cut’/‘torn’ phenotype, after 8 hours at 36°C (Figure 2A–2B). The *rqh1Δ* and *top3Δ rqh1Δ* mutants display similar but less pronounced defects in chromosome segregation compared to the *top3-105* mutant (p = 0.024 and p = 0.00023) (Figure 2A–2B and Table S1). Deletion of *rad51* rescues the severe mitotic defects of the *top3-105* mutant (p = 0.0046). However, the *top3-105 rad51Δ* double mutant and the *rad51Δ* single mutant both still display moderate defects in chromosome segregation. Moreover, deletion of *rad51* does not affect the chromosome segregation defects of the *rqh1Δ* mutant, as the *rqh1Δ rad51Δ* double mutant displays similar levels of mitotic defects as the *rqh1Δ* and *rad51Δ* single mutants (Figure 2A–2B and Table S1). Therefore, it seems that Top3, Rqh1 and Rad51 are all important for normal mitotic chromosome segregation.

### Top3 is highly enriched at intergenic regions, sub-telomeres, and centromeres

**Figure 3:**
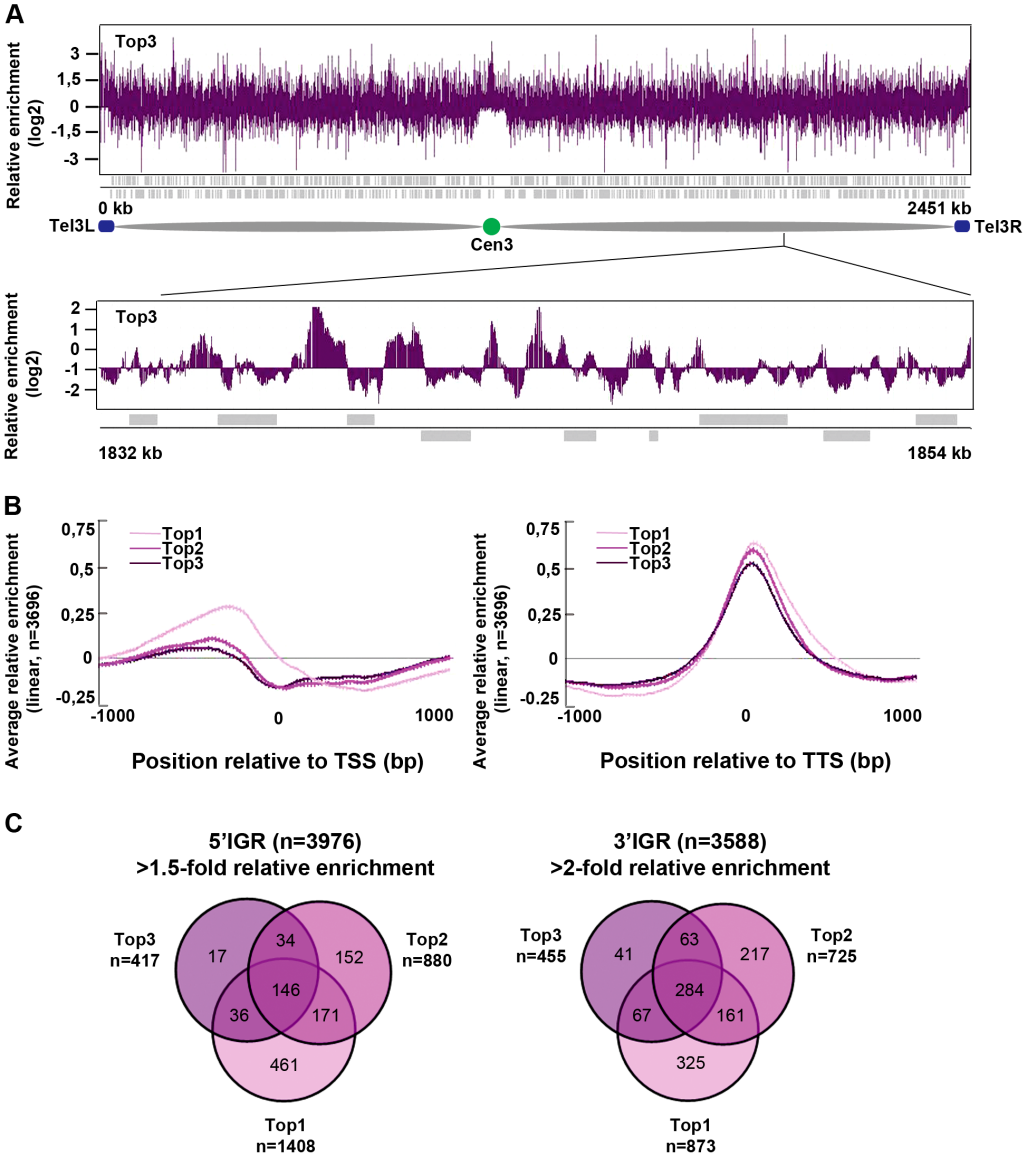
Top3 is enriched at IGRs, centromeres, and sub-telomeric regions. (A) ChIP-chip relative enrichment of Top3-myc along chromosome III at 30°C. The schematic picture shows the approximate position of the centromere and the subtelomeric regions. Telomeric repeats are not represented on the array. (B) Moving average for the relative enrichment of Top3, Top2 and Top1 after alignment of genes at the TSS and TTS, respectively. Error bars represent 99% confidence intervals. The bottom bar illustrates statistical significance by t-tests for the difference between the graphs at each point using a continuous spectrum from black (p = 1) via red to yellow (p = 0). (C) Overlaps between 5′IGRs with an average >1.5-fold and 3′IGRs with an average >2-fold relative enrichment of Top3, Top2 and Top1, respectively. The overlaps are statistically significant (p<0.001) by pair-wise hyper-geometric distribution tests. All data is an average of two independent experiments.

Next, we investigated the genome-wide relative enrichment of Top3 by chromatin immunoprecipitation (ChIP) of c-Myc epitope tagged Top3 expressed from the endogenous locus and hybridization to high-resolution tiling microarrays (ChIP-chip). Along the euchromatic chromosome arms high relative enrichment of Top3 is preferentially found at intergenic regions (IGRs), while it is generally depleted from open reading frames (ORFs) (Figure 3A). Top3 occupancy is also found at sub-telomeric regions, including the rDNA clusters at the left and right sub-telomeric regions of chromosome III (*tel3L* and *tel3R*) (Figure 3A and Figure S1A–S1B). Telomeric repeats are not represented on the array and therefore telomere occupancy could not be investigated. Moreover, we observed a consistently high relative enrichment of Top3 at centromeres (Figure 3A and Figure S1A–S1B). Next, we determined the genome-wide average levels of Top3 when genes are aligned at the transcription start site (TSS) and transcription termination site (TTS), respectively. Top3 display high average relative enrichment at the TTS, but depletion from the TSS and the ORF (Figure 3B). There is also a small average enrichment of Top3 just upstream of the TSS, where gene promoters are generally localized. Overall, this binding pattern is similar to that of Top1 and Top2. Depletion of Top3 from the TSS and ORF as well as enrichment at the TTS is more pronounced for strongly transcribed genes, whereas Top3 occupancy at promoter regions is preferentially found at non-transcribed and weakly transcribed genes (Figure S1C). Looking at individual genes, we identified 233 5′IGRs that display a >1.5-fold and 455 3′IGRs that display a >2-fold average relative enrichment of Top3. These overlap significantly (p<0.001) with 5′ and 3′IGRs that are enriched for Top1 and Top2 (p<0.001 for all pair wise comparisons) (Figure 3C). Thus, similar to Top1 and Top2, Top3 preferentially binds to intergenic regions along the euchromatic chromosome arms. In addition, Top3 is enriched at sub-telomeres and centromeres.

### Top3 and gene transcription

**Figure 4:**
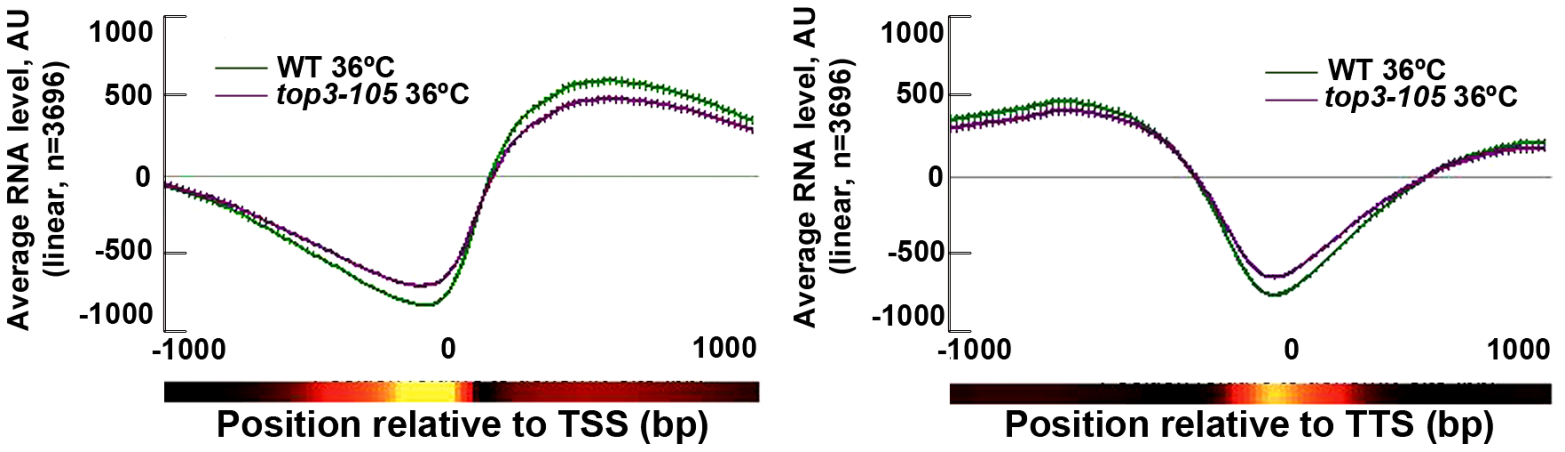
Top3 has small effects on gene transcription. Average RNA levels in wild type and the *top3-105* mutant after 8 hours at 36°C when genes are aligned at the TSS and TTS, respectively. Error bars represent 99% confidence intervals. The bottom bar illustrates statistical significance for the difference between the graphs at each point using a continuous spectrum going from black (p = 1) via red to yellow (p = 0). All data is an average of two independent experiments.

*S. pombe* Top1 and Top2 have previously been implicated in various stages of transcription [30]–[31]. We investigated the genome-wide transcription levels in the *top3-105* mutant by total RNA extraction, reverse transcription and hybridization to tiling microarrays. After 8 hours at 36°C, there is a small reduction in the genome-wide average RNA levels from ORFs in the *top3-105* mutant compared to wild type (Figure 4). However, after filtering out non-transcribed genes (AU<100 in both wild type and the *top3-105* mutant) we only identified 118 (106 protein coding) genes that displayed a >1.5-fold decrease of RNA levels in the *top3-105* mutant. Moreover, there are also 299 (211 protein coding) genes that display a >1.5-fold increase of RNA levels in the *top3-105* mutant. Thus, the *top3-105* mutant displays minor changes in RNA levels, associated with both up- and down-regulation of gene transcription.

### Top3 and CENP-A^Cnp1^ co-localize at centromeres and 5′IGRs

**Figure 5:**
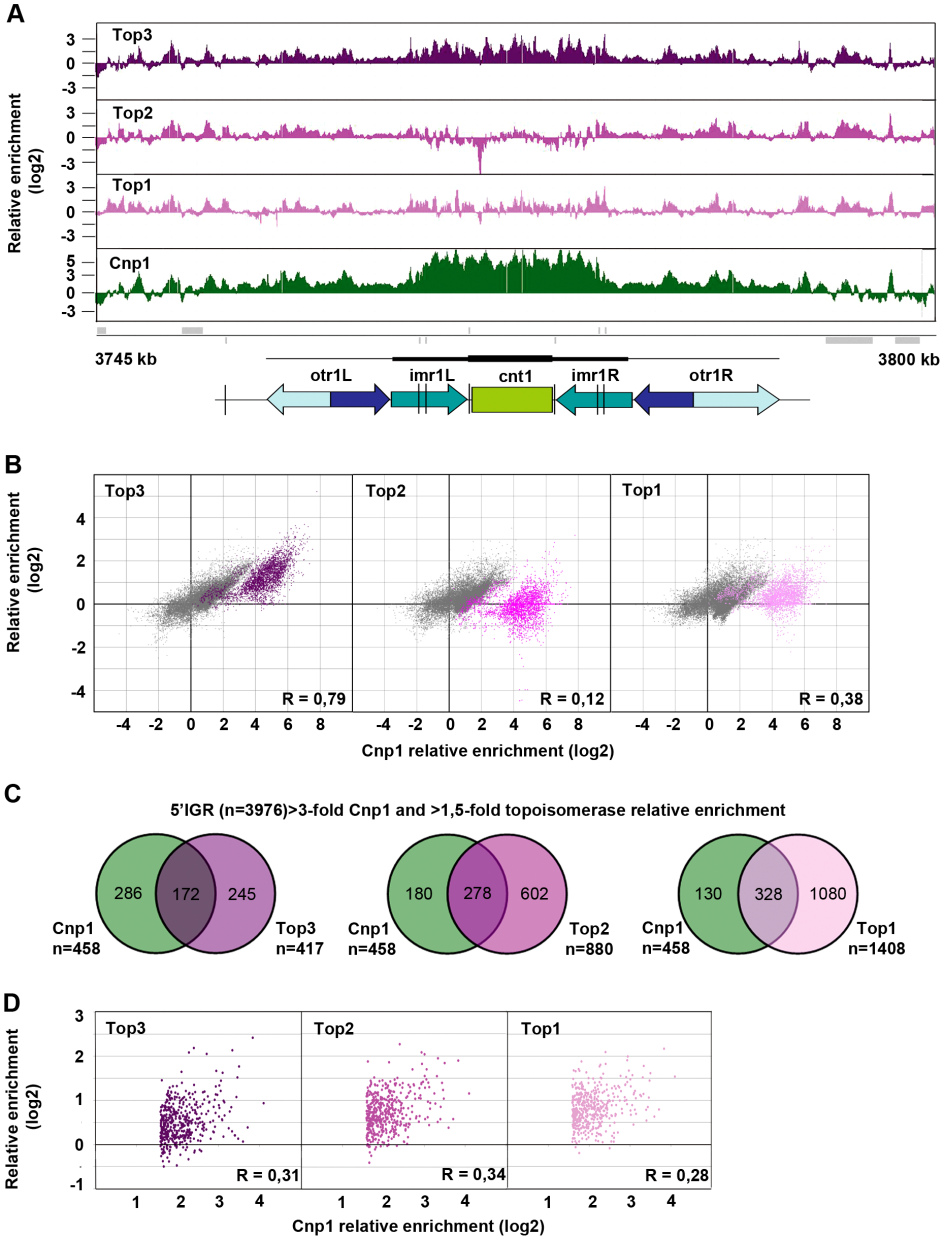
Top3 and CENP-A^Cnp1^ occupancies are positively correlated. (A) ChIP-chip relative enrichment of Top3-myc, Top2-myc, Top1-myc and CENP-A^Cnp1^ along centromere I at 30°C. Grey boxes represent genes. A schematic representation where arrows represent repeat elements and black lines represent tRNA genes is shown. The vertical arrow indicates the position of primers used in D-F. The scale bar represents 2.5 kb. (B) Density scatter plot for all centromeric probes showing the correlations between the relative enrichment of Cnp1 and Top3, Top2, and Top1, respectively. Probes corresponding to central cores are shown in color. Pearson's correlation coefficients for all centromeric probes (R) are shown. (C) Overlaps between 5′IGRs with an average >3-fold relative enrichment of Cnp1 and >1.5-fold relative enrichment of Top3, Top2 and Top1, respectively. The overlaps are statistically significant (p<0.001) by pair-wise hyper-geometric distribution tests. (D) Scatter plot for 5′IGRs with high (>1.5-fold) relative enrichment of Cnp1 showing the correlations between the average relative enrichment of Cnp1 and Top3, Top2, and Top1, respectively. Pearson's correlation coefficients (R) are shown. All data is an average of two independent experiments.

Top3 occupancy at centromeres is most pronounced at the central domain, consisting of the central core (*cnt*) and the innermost repeat (*imr*) regions, where there is 4–8 fold enrichment of Top3 relative to the rest of the genome (Figure 5A and Figure S2). Interestingly, both Top1 and Top2 occupancies are relatively low at this region. The central domain, and particularly the *cnt* region, is also highly enriched for CENP-A^Cnp1^. Centromeric CENP-A^Cnp1^ occupancy displays a strong positive correlation with Top3 occupancy, but no correlation with Top2 and a weaker positive correlation with Top1 occupancy (Figure 5B). In fact, there is a subset of probes forming a separate cluster in the scatter plot for which there is a unique positive correlation with Top3 enrichment as opposed to Top1 and Top2 enrichment. This cluster corresponds to the majority of the *cnt* probes, for which the relative enrichment of CENP-A^Cnp1^ is distinctively high. In *S. pombe*, it has previously been shown that CENP-A^Cnp1^ also localizes to gene promoters [60]. 5′IGRs that display a >3-fold average relative enrichment of CENP-A^Cnp1^ significantly overlap with those that display a >1.5-fold average relative enrichment of Top3 (p<0.001) (Figure 5C). However, as expected from the similar bindings of Top1 and Top2 to promoter regions, there are also significant overlaps with those that display a >1.5-fold average relative enrichment of Top2 and Top1, respectively (p<0.001 for pair-wise comparisons). Moreover, average CENP-A^Cnp1^ occupancies at these 5′IGRs display a weaker positive correlation with Top3 occupancy, as well as with Top2 and Top1 occupancies (Figure 5D). Thus, Top3 co-localizes with CENP-A^Cnp1^ at both centromeres and 5′IGRs, but centromeres are distinctive in that they display a unique correlation between Top3 occupancy and high levels of CENP-A^Cnp1^ occupancy, which is not seen for Top1 and Top2.

### Increased levels and redistribution of non-centromeric CENP-A^Cnp1^ at 5′IGRs in the *top3-105* mutant

**Figure 6:**
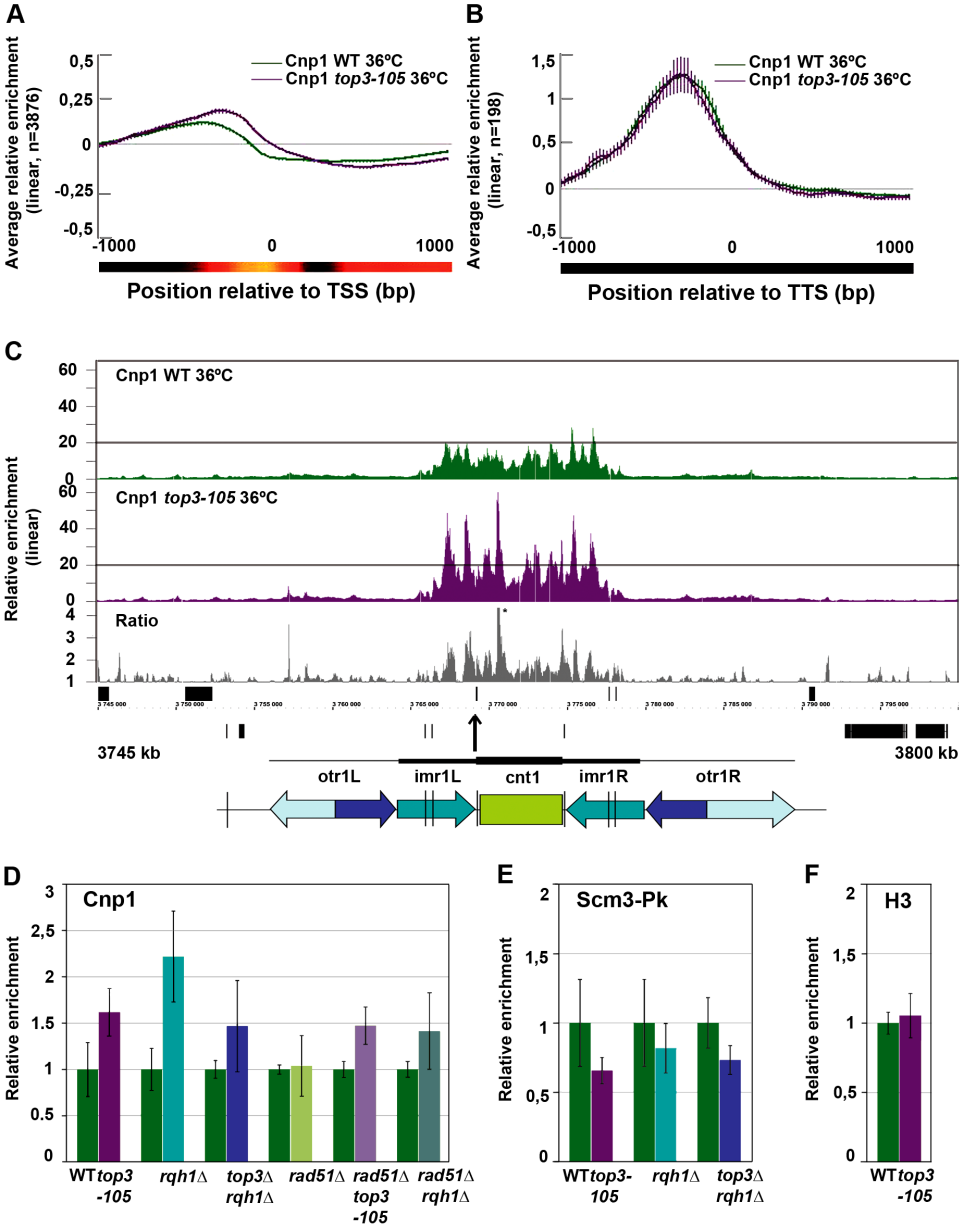
Top3 and Rqh1 affect CENP-A^Cnp1^ enrichment at 5′IGRs and centromeres. (A) Moving average for the relative enrichment of CENP-A^Cnp1^ in wild type and the *top3-105* mutant after alignment of genes at the TSS. Error bars represent 99% confidence intervals. The bottom bar illustrates statistical significance for the difference between the graphs at each point using a continuous spectrum going from black (p = 1) via red to yellow (p = 0). (B) Same as in A for 198 genes with >1.5-fold relative enrichment of CENP-A^Cnp1^ in wild type. (C) ChIP-chip relative enrichment of CENP-A^Cnp1^ in wild type and the *top3-105* mutant and the ratio between these along centromere I after 8 hours at 36°C. Grey boxes represent genes. A schematic representation where arrows represent repeat elements and black lines represent tRNA genes is shown. A * indicates that the peak is higher than the maximum value of the axis. (D) ChIP-qPCR relative enrichment of CENP-A^Cnp1^ in wild type and the indicated mutants after 8 hours at 36°C. (E) ChIP-qPCR average relative enrichment of HJURP^Scm3^-Pk/V5 in wild type and the indicated mutants after 8 hours at 36°C. (F) ChIP-qPCR average relative enrichment of H3 in wild type and the *top3-105* mutant after 8 hours at 36°C. ChIP-qPCR was performed using triplicate samples in two independent experiments. Relative enrichment at the *cnt* region of chromosome I was calculated using the ddCt method, normalizing to ChIP input and *act1*. Samples were also normalized to the average of the wild type samples in each experiment. Error bars represent the standard deviations between six samples.

Next, we used ChIP-chip to investigate the genome-wide relative enrichment of CENP-A^Cnp1^ in the *top3-105* mutant after 8 hours at 36°C. There is a small increase in the genome-wide average relative enrichment of CENP-A^Cnp1^ at promoter regions in the *top3-105* mutant compared to wild type (Figure 6A). However, there is no difference in the average relative enrichment of CENP-A^Cnp1^ at the 198 5′IGRs that display a high (>3-fold) relative enrichment of CENP-A^Cnp1^ in wild type (Figure 6B). Among these sites, there are 13 that display a >1.5-fold increase and 54 that display a >1.5-fold decrease in the relative enrichment of CENP-A^Cnp1^ in the *top3-105* mutant. Moreover, we identified 85 5′IGR that are not enriched for CENP-A^Cnp1^ in wild type, but display a >1.5-fold increase resulting in a rather high (>1.5-fold) relative enrichment of CENP-A^Cnp1^ in *top3-105* mutant. Thus, there is both an overall increase in the average levels and a redistribution of CENP-A^Cnp1^ at promoter regions in the *top3-105* mutant.

### Increased levels of centromeric CENP-A^Cnp1^ and decreased levels of HJURP^Scm3^ in *top3-105* and *rqh1Δ* mutants

At centromeres, where there is a unique overlap between Top3 and CENP-A^Cnp1^ occupancy, there is a consistent increase in CENP-A^Cnp1^ enrichment at the central domains in the *top3-105* mutant compared to wild type after 8 hours at 36°C (Figure 6C and Figure S3). This does not seem to be due to indirect effects by changes in transcription, since no genes known to be involved in CENP-A^Cnp1^ assembly or disassembly were found among those that displayed >1.5-fold increase or decrease in RNA levels in the *top3-105* mutant (Table S2). We also investigated the total amounts of CENP-A^Cnp1^ protein by acid extraction of histones from chromatin and immunoblotting using strains expressing FLAG epitope tagged CENP-A^Cnp1^ from the endogenous locus. In agreement with the ChIP data, there is an increase in the total levels of CENP-A^Cnp1^ protein associated with chromatin in the *top3-105* mutant compared to wild type (Figure S4). The soluble fraction of CENP-A^Cnp1^ is small in both wild type and mutants, but may be slightly higher in the mutant. Using ChIP and qPCR we confirmed that there is an increase in the relative enrichment of CENP-A^Cnp1^ at the *cnt* region of chromosome I in the *top3-105* mutant compared to wild type after 8 hours at 36°C (p = 0.0030) (Figure 6D). This phenotype is found also in the *rqh1Δ* mutant and the *top3Δ rqh1Δ* mutant (p = 0.00026 and p = 0.045, respectively). The *rad51Δ* single mutant on the other hand displays similar levels of CENP-A^Cnp1^ compared to wild type in this region. Moreover, deletion of *rad51* does not suppress the increase in CENP-A^Cnp1^ enrichment in the *top3-105* and *rqh1*Δ mutants, as the *rad51Δ top3-105* and *rad51Δ rqh1Δ* double mutants still display increased relative enrichment of CENP-A^Cnp1^ compared to wild type at the *cnt* region of chromosome I (p = 0.00035 and p = 0.037) (Figure 6D). Thus, Top3 and Rqh1 normally limit the levels of CENP-A^Cnp1^ by a mechanism that is largely independent of their role in HR downstream of Rad51.

Next, we investigated whether the altered levels of CENP-A^Cnp1^ is associated with changes in the relative enrichment of the CENP-A^Cnp1^-specific chaperone HJURP^Scm3^ by ChIP-qPCR using strains expressing Pk/V5 epitope tagged HJURP^Scm3^ from the endogenous locus. Surprisingly, the enrichment of HJURP^Scm3^ at the *cnt* region of chromosome I is reduced in the *top3-105*, *rqh1Δ* and *top3Δ rqh1Δ* mutants compared to wild type after 8 hours at 36°C (p = 0.028, p = 0.24 and p = 0.011, respectively) (Figure 6E). Thus, Top3 and Rqh1 have the opposite effect on the levels of HJURP^Scm3^ compared to the levels of CENP-A^Cnp1^ in this region. Last, we tested if the altered levels of CENP-A^Cnp1^ at centromeres were associated with altered levels of histone H3 in this region. However, there was no significant difference in the relative enrichment of histone H3 at the *cnt* region of chromosome I in the *top3-105* mutant. Thus, Top3 and Rqh1 affect centromeric chromatin in a way that specifically limits the levels of CENP-A^Cnp1^ and promotes association of HJURP^Scm3^ at central domains.

## Discussion

### Top3 and Rqh1 are required for accurate chromosome segregation

The *top3-105* mutant carries an A762G single base pair substitution resulting in a Tyr209Cys amino acid change. This residue is conserved in *S. cerevisiae* Top3 as well as in metazoan Top3α, and resides in an important region involved in conformational changes upon DNA binding [57]. Similar to other thermo-sensitive *top3* mutants, the *top3-105* mutant displays impaired growth, altered nuclear morphology and various defects in chromosome segregation soon after a shift to the restrictive temperature [46], [53]. Deletion of *top3* is known to cause very severe nuclear and mitotic defects resulting in cell death after just a few generations [51]–[52] Since the lethality can be suppressed by deletion of *rqh1* it is hypothesized that these extreme nuclear defects are caused by rapid accumulation of unresolved Rqh1-dependent HR intermediates that prevent chromosome segregation and ultimately causes lethality. However, both the *rqh1Δ* single mutant and the *top3Δ rqh1Δ* double mutant still display moderate mitotic defects, indicating that there may be additional roles for the Top3-Rqh1 complex in chromosome segregation. Rad51 has previously been shown to be important for normal mitotic chromosome segregation and the *rad51Δ* mutant displays moderate defects in chromosome segregation [58]. Deletion of *rad51* also suppresses the severe chromosome segregation defects of the *top3-105* mutant, but the *top3-105 rad51Δ* double mutant also still display moderate defects in chromosome segregation. Deletion of *rad51* has no effect on the mitotic defects in the *rqh1Δ* mutant, as the *rqh1Δ rad51Δ* double mutant display similar levels of chromosome segregation defects as the *rqh1Δ* and *rad51Δ* single mutants. Thus, it seems that Top3, Rqh1 and Rad51 are all important for normal mitotic chromosome segregation.

### Top3 is the major topoisomerase at centromeric central domains

The genome-wide localization of Top3 reveals high relative enrichment at IGRs, towards subtelomeric regions and at centromeres. The binding pattern for Top3 at IGRs is similar to those of Top1 and Top2, possibly indicating that all tree topoisomerases have overlapping functions at promoters and TTSs, such as maintenance of 5′ and 3′ nucleosome depleted regions (NDRs) important for transcription [30]–[31]. However, we did not find any major effects on transcription for the *top3-105* mutant, and Top3 association at promoters is mostly seen at non-transcribed genes. The relative enrichment of Top3 is also high towards subtelomeric regions, including the rDNA clusters at *tel3L* and *tel3R*. This is in agreement with the role of *S. pombe* Top3 and Rqh1 in replication recombination at rDNA and telomere repeats [46]–[47], [61]. Surprisingly, Top3 is also enriched at centromeres and particularly at centromeric central domains, where there is no pronounced enrichment of Top1 and Top2. Thus, Top3 is the major topoisomerase present at centromeric central domains and may have a unique function in this region.

### Top3 and Rqh1 affect the relative enrichment of CENP-A^Cnp1^

The chromatin structure found at centromeric central domains is unique in that it contains high levels of the histone H3 variant CENP-A^Cnp1^. Interestingly, Top3 occupancy displays a unique positive correlation with CENP-A^Cnp1^ occupancy in these regions, and especially at the *cnt* regions where the enrichment of CENP-A^Cnp1^ is particularly high. This led us to investigate if Top3 has an effect on CENP-A^Cnp1^ nucleosomes in this region. Intriguingly, the relative enrichment of CENP-A^Cnp1^ at central domains is increased both in the *top3-105* mutant, the *rqh1Δ* mutant and the *top3Δ rqh1Δ* double mutant. This suggests that the activity of the Top3-Rqh1 complex normally limits the levels of CENP-A^Cnp1^ in this region. Intriguingly, the altered structure and/or dynamics of centromeric CENP-A^Cnp1^-containing chromatin may contribute to the chromosome segregation defects seen in the *top3-105, rqh1Δ* and *top3-105 rqh1Δ* mutants. However, this new role is clearly not the cause of the extremely severe chromosome segregation defects and lethality in the *top3Δ* mutant, as the *top3Δ rqh1Δ* mutant is viable while still retaining this phenotype. As previously described, the lethality of the *top3Δ* mutant likely depends on accumulation of unresolved Rqh1-dependent recombination intermediates, which are probably independent of changes in centromeric chromatin.

Interestingly, CENP-A^Cnp1^ has also been shown to be associated with gene promoters [60]. Like for centromeric CENP-A^Cnp1^, there is an overall increase in the relative enrichment of CENP-A^Cnp1^ at promoter regions in the *top3-105* mutant. In addition, there is a partial redistribution of non-centromeric CENP-A^Cnp1^. Thus, it is clear that Top3 also affects the dynamics of CENP-A^Cnp1^ outside of centromeres. However, since Top3, Top2 and Top1 are all enriched at promoter regions, the dynamics of CENP-A^Cnp1^ outside centromeres is likely to depend on all three topoisomerases, making the situation complex.

### Top3 may affect CENP-A^Cnp1^ nucleosome dynamics by regulating centromeric DNA topology

The most established role of the Top3-Rqh1 complex is in HR, where they act downstream of Rad51. One possibility is that the effects on CENP-A^Cnp1^ enrichment relates to the role of Top3 and Rqh1 in this pathway. Rad51 is required for accurate chromosome segregation and has previously been shown to suppress chromosomal rearrangements at centromeres [58], [62]. Moreover, it was recently hypothesized that HR could be involved in higher-order organization of *S. pombe* centromeres [63]. However, increased relative enrichment of CENP-A^Cnp1^ is still seen in the *top3-105 rad51Δ* and *rqh1Δ rad51Δ* double mutants, but not in the *rad51Δ* single mutant. Therefore, the role of the Top3-Rqh1 complex in limiting the levels of CENP-A^Cnp1^ seems largely independent of Rad51-dependent HR. Chromosome segregation defects are on the other hand also seen in the *rad51Δ* single mutant, indicating that HR may somehow be important for proper chromosome segregation. Thus, chromosome segregation defects in *top3* and *rqh1* mutants could originate both from defects in HR and from altered levels of CENP-A^Cnp1^ at centromeres. Another possibility is that the Top3-Rqh1 complex has an indirect effect on CENP-A^Cnp1^ dynamics due to altered transcription of genes involved in CENP-A^Cnp1^ nucleosome dynamics. However, we did not find any significant change in transcription for any of the genes currently known to be involved in CENP-A^Cnp1^ dynamics. A third possibility is that Top3 and Rqh1 affects the stability of the CENP-A^Cnp1^ protein. The total amount of CENP-A^Cnp1^ present in cells is increased in the *top3-105* mutant compared to wild type. However, this seems to mostly relate to an increase in the total levels of CENP-A^Cnp1^ associated with chromatin, while the amount of soluble CENP-A^Cnp1^ is low in both wild type and mutant. In budding yeast, it has been shown that the amount of soluble CENP-A is tightly controlled by rapid proteolysis, while nucleosome assembly at centromeres stabilizes the protein [64]. In agreement, reduced levels of CENP-A^Cnp1^ at centromeres have previously been associated with a reduction of the total CENP-A^Cnp1^ protein levels found in cells [65]. Thus, increased protein levels of CENP-A^Cnp1^ is in agreement with increased levels of CENP-A^Cnp1^ nucleosomes at centromeres, and it seems less likely that impaired Top3 function would stabilize soluble CENP-A^Cnp1^. Instead, we suggest that Top3 together with Rqh1 affect the assembly and disassembly of CENP-A^Cnp1^ nucleosomes by regulating centromere DNA topology.

Nucleosome assembly and disassembly are intimately linked to DNA topology and DNA topoisomerases have previously been shown to affect the assembly of canonical H3 nucleosomes [27]–[31]. In *S. pombe*, Top1 and Top2 have been implicated in disassembly of H3 nucleosomes at 5′ and 3′ IGRs [30]–[31]. Here, removal of negative supercoiling by Top1 and Top2 is hypothesized to stimulate nucleosome disassembly mediated by the chromatin remodeler Hrp1. Centromeres are likely to be topologically constrained regions where nucleosome dynamics are highly dependent on DNA topology and topoisomerases. Since Top3 is highly enriched at central domains as compared to the other topoisomerases, nucleosome dynamics in this region likely depends particularly on Top3. Top3 is unique in that it preferentially removes negative supercoiling. Thus, the activity of Top3 should have a negative effect on assembly of CENP-A^Cnp1^ nucleosomes with left-handed wrapping of DNA. This could be a way of controlling and fine-tuning the assembly of CENP-A^Cnp1^ nucleosomes mediated by factors such as HJURP^Scm3^. In the *top3-105* mutant, a shift towards a state of more negative supercoiling would result in increased stability and facilitated assembly of CENP-A^Cnp1^ nucleosomes, altering the dynamics of CENP-A^Cnp1^ nucleosomes. In agreement with increased relative enrichment of CENP-A^Cnp1^ also in the *rqh1Δ* mutant, efficient relaxation of negative supercoils by Top3 has been shown to be dependent on RecQ helicases [41], [66]. HJURP^Scm3^ associates with centromeric chromatin during most of the cell cycle, independently of CENP-A^Cnp1^, but dissociates from centromeres right after assembly of newly synthesized CENP-A^Cnp1^ in the G2 phase of the cell cycle [8]–[9], [67]. Reduced levels of HJURP^Scm3^ at centromeres in the *top3-105* and *rqh1Δ* mutants may thus reflect facilitated loading of CENP-A^Cnp1^ from the pre-nucleosomal complex onto centromeric DNA and a more rapid dissociation of HJURP^Scm3^.

### An alternative or additional role in centromeric transcription

In addition to nucleosome dynamics, DNA topology is also known to be important for transcription. Recent studies have shown that transcription is permissive also at the CENP-A^Cnp1^ containing centromeric central domains in fission yeast [60]. Although the exact function is unclear, carefully modulated centromeric transcription has been suggested to play a role in formation of kinetochores as well as assembly of CENP-A^Cnp1^ nucleosomes [68]. In support, factors known to be important for transcription have been implicated in CENP-A^Cnp1^ assembly [65], [69]–[70]. Thus, it is possible that Top3 as the main DNA topoisomerase at centromeric central domains affects transcription-coupled CENP-A^Cnp1^ assembly. However, this is not mutually exclusive with a direct effect on CENP-A^Cnp1^ nucleosome assembly.

### Is Top3 a factor that controls the structure of CENP-A^Cnp1^ nucleosomes?

The molecular architecture of CENP-A nucleosomes is a subject of debate. Some studies suggest that CENP-A, H4, H2A and H2B form hemisomes with right-handed wrapping of DNA [20], [22]–[24]. In this case, due to opposite wrapping of the DNA double helix, preferential relaxation of negative supercoils by Top3 should increase the stability of CENP-A^Cnp1^ hemisomes. In this model, impaired Top3 activity in the *top3* and *rqh1* mutants may result in a shift from assembly of right-handed CENP-A^Cnp1^ hemisomes toward assembly of left-handed octameric CENP-A^Cnp1^ nucleosomes at centromeres, thus giving increased levels of CENP-A^Cnp1^. Such a structural transition has previously been observed upon ectopic incorporation of *S. cerevisiae* CENP-A^Cse4^ at non-centromeric loci [24]. Recent studies have also suggested that CENP-A nucleosomes may cycle between octameres and tetramers during the cell cycle [25]–[26]. In this case, the Top3-Rqh1 complex may affect one or both species. Interestingly, it was also shown human HJURP and budding yeast Scm3 associates with centromeres specifically during formation of CENP-A hemisomes [25]–[26]. Thus, reduced levels of Scm3 at centromeres in *top3* and *rqh1* mutants can thus also be reconciled with a structural change in CENP-A chromatin during some part of the cell cycle. Moreover, the fact that CENP-A^Cnp1^ levels are increased, while H3 levels remains the same in the Top3 mutant, would be consistent with a structural change specific for CENP-A^Cnp1^ nucleosomes.

In conclusion, we found that *S. pombe* Top3 displays a unique enrichment at centromeres where it affects centromeric chromatin in a way that limits the levels of CENP-A^Cnp1^. We suggest that the Top3-Rqh1 complex has an important role in regulating centromeric DNA topology, thereby affecting CENP-A^Cnp1^ nucleosome dynamics and perhaps the structure CENP-A^Cnp1^ nucleosomes. Thus, the Top3-Rqh1 complex may be part of the intricate network of factors and pathways that comes together to carefully regulate CENP-A^Cnp1^ nucleosome dynamics. This function could contribute to the observed chromosome segregation defects in *top3* and *rqh1* mutants.

## Materials and Methods

### Fission yeast strains and methods

Standard procedures for genetic manipulation and growth of *S. pombe* were used [71]. The strain expressing Pk/V5 epitope tagged HJURP^Scm3^ was a gift from Professor M. Yanagida, the thermo-sensitive *top3-105* mutant and the *rad51Δ* mutant were acquired from the Yeast Genetic Resource Center (YGRC). The *rqh1*Δ mutant was a gift from S-W. Wang. Strains used in this study are listed in Table S3.

### Sequencing of the *top3-105* mutation

Genomic DNA was isolated as previously described, except that cell walls were digested with 0.4 mg/ml zymolyase 100T (USBiological) and RNA was digested with 10 µg/ml RNase (Roche) for 60 minutes at 37°C [71]. The *top3* open reading frame was amplified by PCR and the purified PCR product was sent to Eurofins MWG Operon for custom DNA sequencing. Primers used are listed in Table S4. Protein sequence alignments were generated using ClustalW2 (EMBL-EBI, http://www.ebi.ac.uk/Tools/msa/clustalw2/).

### Spotting and analysis of growth kinetics

Cells grown to log-phase at 25°C were spotted in 5-fold serial dilutions and incubated at 25°C, 30°C or 36°C. Cells were grown to early log-phase at 25°C and then shifted to 36°C for 9 hours, while determining cell density hourly using a microscope counting chamber.

### Immunofluorescence microscopy

Immunofluorescence microscopy was performed as previously described with the following details and alterations [72]. Cells were grown to mid-log phase first at 25°C and then at 36°C for 8 hours. Cells were fixed in 3.7% formaldehyde and 0.2% glutaraldehyde for 60 minutes before digestion with 0.5 mg/ml zymolyase 100T (US Biological) for 70 minutes at 37°C. Cells were incubated with 1∶80 dilution of TAT1 mouse anti-tubulin serum (a gift from K. Gull) over night and then with 1∶100 dilution of FITC-conjugated goat anti-mouse (F-1010, Sigma) over night. Cells were stained with 0.2 µg/ml 4′.6-diamidino-2-phenylindole (DAPI) and mounted on poly-L-lysine-coated microscopy slides (LabScientific) using Vectashield mounting media (Vector laboratories). Cells were examined by fluorescence microscopy using a Zeiss Axioplan 2 (Carl Zeiss) equipped with a Plan-Neofluar 63X/1.25 PH3 oil objective (Carl Zeiss), an ORCA-100 CCD camera (Hamamatsu Photonix) and Openlab 5.0.2 software (Improvision). P values for comparing chromosome segregation defects were generated using a two-tailed Fishers exact test.

### Chromatin Immunoprecipitation (ChIP) and preparation for tiling microarrays

ChIP was performed as previously described with the following details and alterations [73]. Cells were grown to mid-log phase at 30°C or first at 25°C and then at 36°C for 8 hours. Cells were lysed using a FastPrep-24 homogenizer (MP Biomedicals) with seven 30 second pulses at 6.5 m/s. Chromatin was fragmented using a Vibra-cell VCX 130 sonicator (Sonics) equipped with a 2 mm stepped microtip, set to 40% amplitude with 10 second pulses and 15 second pauses for two minutes. Chromatin fragments were immunoprecipitated with 1 µg 9E10 mouse anti-Myc (M4439 Sigma), 3 µg rabbit anti-H3 (Ab1791 Abcam), 10 µl rabbit anti-Cnp1 antiserum (a gift from R. Allshire) or 3 µg mouse anti-V5 (MCA1360 Serotec). DNA was recovered using QIAquick PCR Purification with Buffer PB (Qiagen). For analysis on GeneChip *S. pombe* Tiling 1.0FR Arrays (Affymetrix) 5 mM dUTP was added to the second round of DNA amplification. Fragmentation, labelling and hybridization were performed by the Affymetrix core facility at Karolinska Institiutet (BEA) using standard protocols (http://www.affymetrix.com).

### Total RNA extraction and preparation for tiling microarrays

Cells were grown to mid-log phase first at 25°C and then at 36°C for 8 hours. Total RNA was extracted using hot acid-phenol and chloroform. Reverse transcription, labeling, fragmentation and hybridization to GeneChip *S. pombe* Tiling 1.0FR Arrays (Affymetrix) was performed by BEA.

### Microarray data analysis

Raw data (.CEL format) was analyzed using Affymetrix Tiling Analysis Software (TAS) v1.1. One sample analysis and linear scaling was used for RNA samples. Two-sample comparison of immunoprecipitated and input samples, with linear scaling and separate quantile normalization was used for ChIP samples. Probe signals were generated using a bandwidth of 100 and assigned to *S. pombe* genome coordinates (Sanger 2007 and Sanger 2004 for centromeres). Browser images were generated using Integrated Genome Browser (IGB) (Affymetrix) and PodBat (www.podbat.org). Podbat was used for averaging signals across ORFs and IGRs, for comparing gene lists and for moving averages (bandwidth 100 probes, step size 20 probes) after alignment of genes at the TSS and TTS based on previous annotations [74]. For statistical analysis, a t-test was performed for each bin under the null hypothesis that there is no difference between two sets. 5′IGRs and 3′IGRs were defined as 500 bp upstream or downstream of the ORF or up until the neighbouring gene if shorter. CENP-A^Cnp1^, Top1 and Top2 ChIP-chip and wild type transcription at 30°C raw data (.CEL format) are from previous studies [30], [75]. The microarray data from this publication have been submitted to the GEO database [http://www.ncbi.nlm.nih.gov/geo/] and assigned the accession number GSE44206.

### Quantitative PCR

Quantitative real-time PCR (qPCR) was performed using the 7500 Fast Real-Time PCR System (Applied Biosystems) and the associated Sequence Detection Software v.1.3 (Applied Biosystems). ChIP relative enrichment was calculated using the ddCt method, normalizing to ChIP input (dCt) and to a control locus (ddCt) (*act1*). For each experiment all samples were also normalized to the average of the wild type. Standard deviations were calculated for the total of six samples from two independent experiments. P values were generated using an unpaired two-sample t-test. Primers used are listed in Table S4.

## Supporting Information

Figure S1 Top3 is enriched at centromeres and sub-telomeric regions. (A) ChIP-chip relative enrichment of Top3-myc along chromosome I at 30°C. The schematic picture shows the approximate position of the centromere and the subtelomeric regions. Telomeres are not represented on the array. (B) Same as above but for chromosome II. (C) Moving average for the relative enrichment of Top3 after alignment of genes with different transcription levels at the TSS and TTS, respectively. Error bars represent 99% confidence intervals. The bottom bar illustrates statistical significance for the difference between very low and very high transcription at each point using a continuous spectrum going from black (p = 1) via red to yellow (p = 0). All data is an average of two independent experiments.
(TIF)

Figure S2 Top3 is enriched at centromeric central domains. (A) ChIP-chip relative enrichment of Top3-myc, Top2-myc, Top1-myc and CENP-A^Cnp1^ along centromere II at 30°C. Grey boxes represent genes. A schematic representation where arrows represent repeat elements and black lines represent tRNA genes is shown. (B) Same as above but for centromere III. All data is an average of two independent experiments.
(TIF)

Figure S3 Top3 affects CENP-A^Cnp1^ enrichment at centromeres. (A) ChIP-chip relative enrichment of CENP-A^Cnp1^ in wild type and the *top3-105* mutant and the ratio between these along centromere II after 8 hours at 36°C. Grey boxes represent genes. A schematic representation of where arrows represent repeat elements and black lines represent tRNA genes is shown. The * indicates that the peak is higher than the maximum value of the axis. (B) Same as above but for centromere III. All data is an average of two independent experiments.
(TIF)

Figure S4 The total amount of CENP-A^Cnp1^ protein associated with chromatin is increased in the *top3-105* mutant. Western blot of FLAG in wild type and the *top3-105* mutant for untagged control strains and strains expressing CENP-A^Cnp1^-FLAG from the endogenous locus. After lysis of cells histones were prepared by acid extraction from the insoluble chromatin faction. The soluble fraction was also included in the experiment. Western blot of histone H3 and actin were used as loading controls.
(TIF)

Table S1 Comparisons of chromosome segregation defects between strains. Lists of p values for pair wise comparisons of chromosome segregation defects between the indicated strains. The p values were generated using a two-tailed Fishers exact test. P values indicating a statistical difference between two strains are marked in bold numbers.
(DOCX)

Table S2 Top3 has no major effects on transcription of genes known to affect nucleosome or CENP-A^Cnp1^ dynamics. List of genes that display >1.5-fold up- or down-regulation of RNA levels in top3-105 compared to WT in the Gene Ontology categories GO:0007059 Chromosome Segregation, GO:0051276 Chromosome organization or GO:0000775 Chromosome, centromeric region (n = 460 genes). RNA levels in wild type and the top3-105 mutant and the ratio between these are shown.
(DOC)

Table S3 List of strains used in this study. Strain names and genotypes for all strains used in this study. The right column indicates in which figures each strain has been used.
(DOC)

Table S4 List of primers used in this study. Sequencing and qPCR primers used in this study. The right column indicates which experiment each strain has been used in.
(DOC)
