# Supplementary figures and images for: DNA Topoisomerase III Localizes to Centromeres and Affects Centromeric CENP-A Levels in Fission Yeast
Source: PLoS Genet. 2013 Mar 14;9(3):e1003371. doi: 10.1371/journal.pgen.1003371 (PMC3597498; doi:10.1371/journal.pgen.1003371)

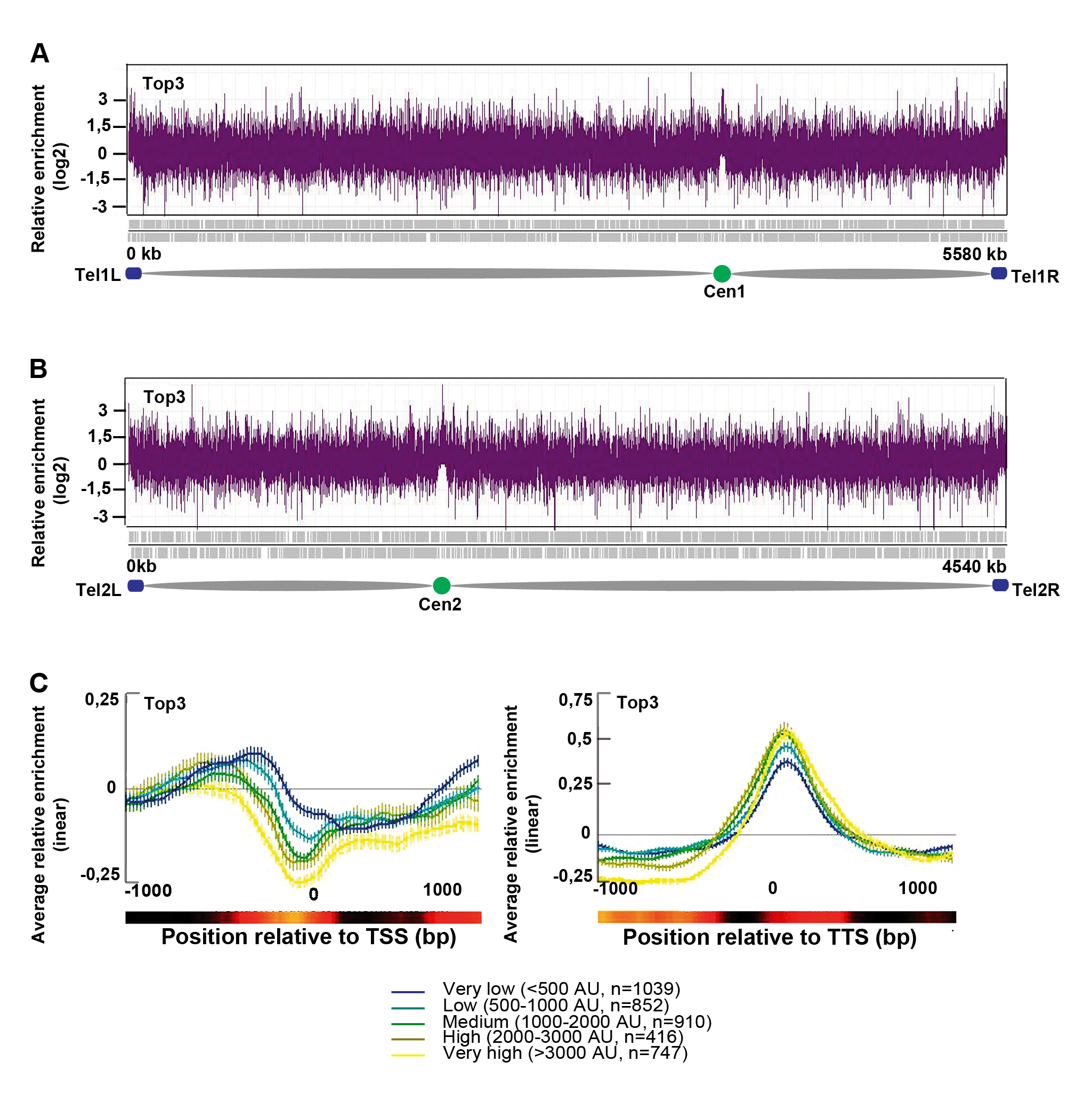

Supplement: Figure S1 — Top3 is enriched at centromeres and sub-telomeric regions. (A) ChIP-chip relative enrichment of Top3-myc along chromosome I at 30°C. The schematic picture shows the approximate position of the centromere and the subtelomeric regions. Telomeres are not represented on the array. (B) Same as above but for chromosome II. (C) Moving average for the relative enrichment of Top3 after alignment of genes with different transcription levels at the TSS and TTS, respectively. Error bars represent 99% confidence intervals. The bottom bar illustrates statistical significance for the difference between very low and very high transcription at each point using a continuous spectrum going from black (p = 1) via red to yellow (p = 0). All data is an average of two independent experiments. (TIF) [file pgen.1003371.s001.tif]

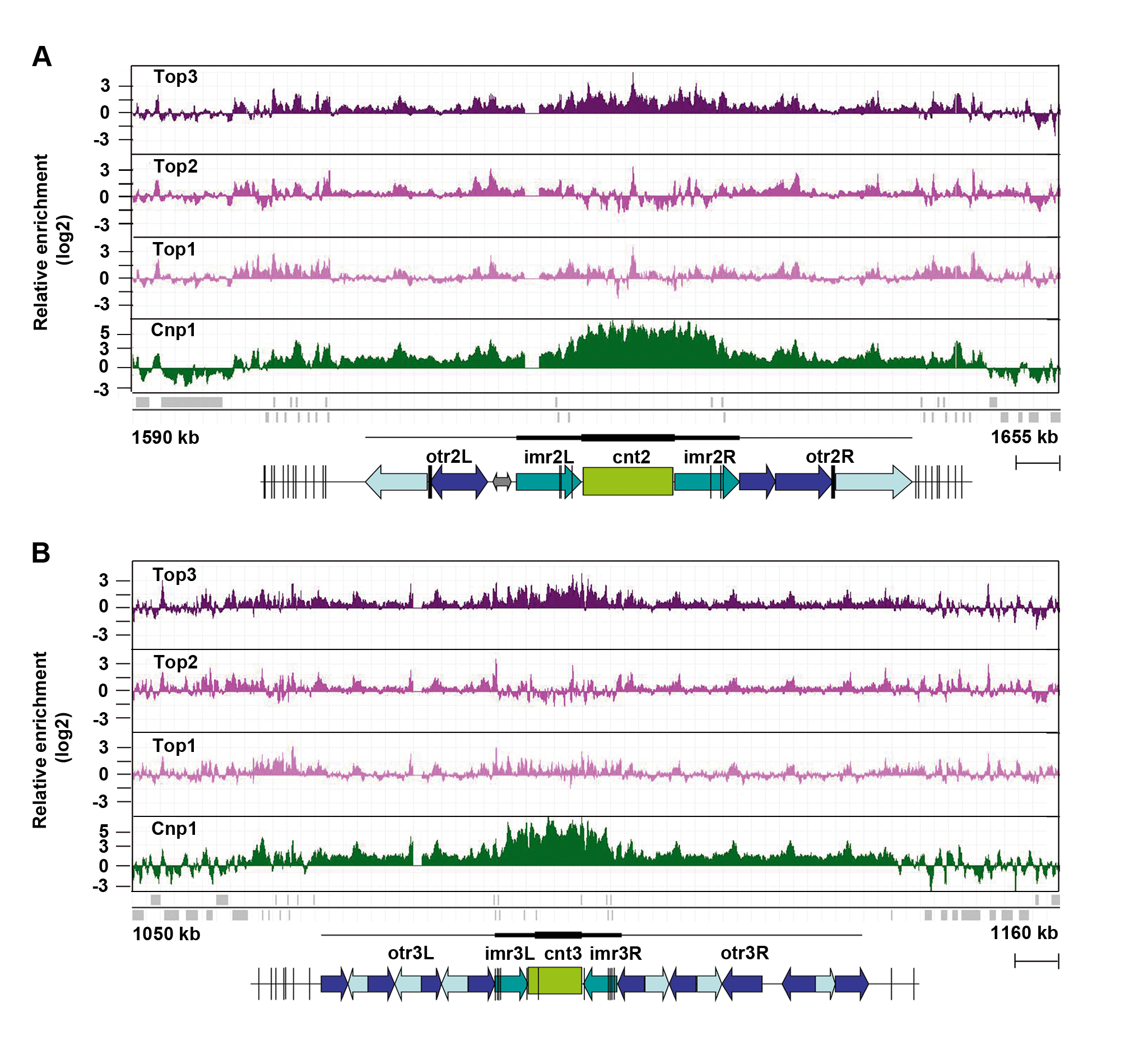

Supplement: Figure S2 — Top3 is enriched at centromeric central domains. (A) ChIP-chip relative enrichment of Top3-myc, Top2-myc, Top1-myc and CENP-ACnp1 along centromere II at 30°C. Grey boxes represent genes. A schematic representation where arrows represent repeat elements and black lines represent tRNA genes is shown. (B) Same as above but for centromere III. All data is an average of two independent experiments. (TIF) [file pgen.1003371.s002.tif]

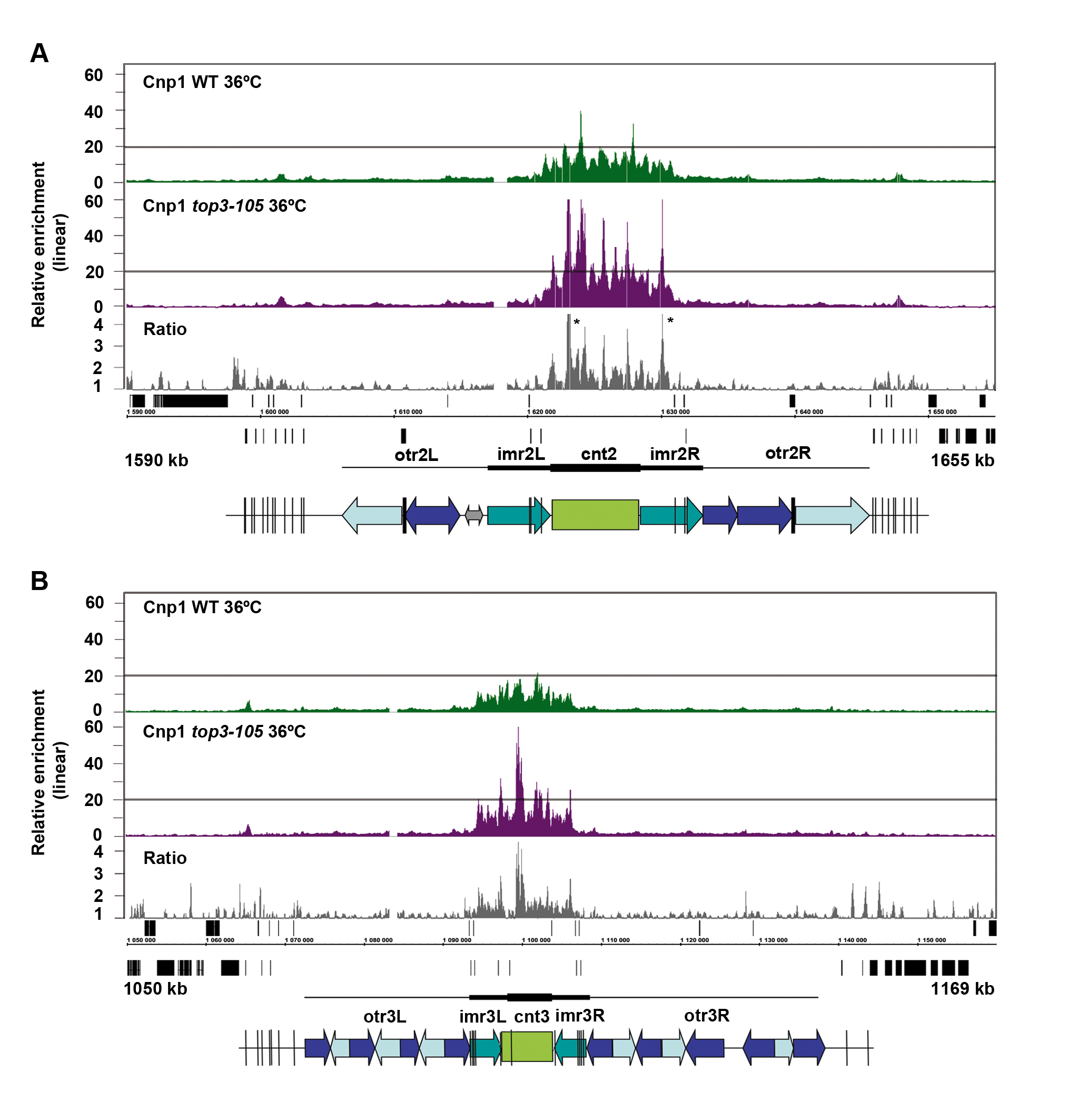

Supplement: Figure S3 — Top3 affects CENP-ACnp1 enrichment at centromeres. (A) ChIP-chip relative enrichment of CENP-ACnp1 in wild type and the top3-105 mutant and the ratio between these along centromere II after 8 hours at 36°C. Grey boxes represent genes. A schematic representation of where arrows represent repeat elements and black lines represent tRNA genes is shown. The * indicates that the peak is higher than the maximum value of the axis. (B) Same as above but for centromere III. All data is an average of two independent experiments. (TIF) [file pgen.1003371.s003.tif]

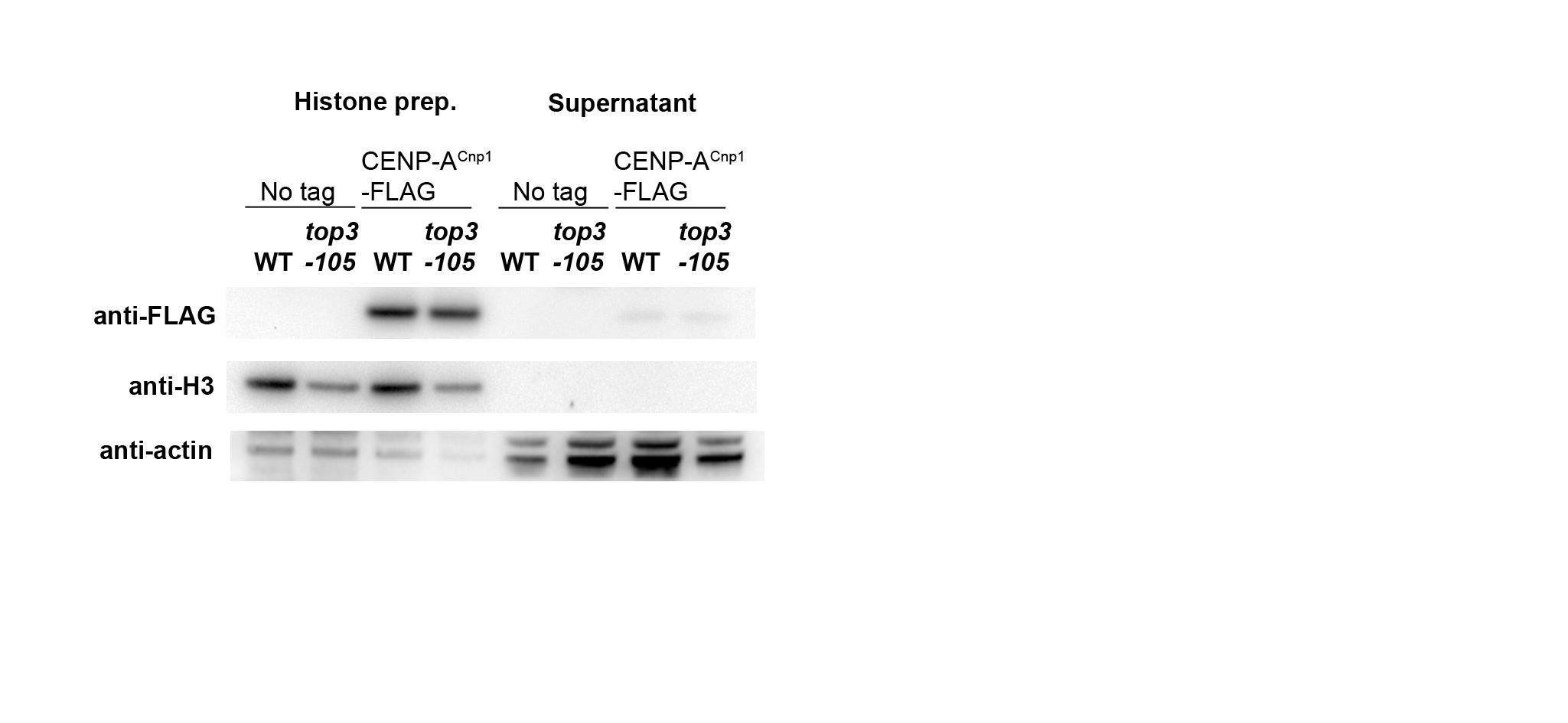

Supplement: Figure S4 — The total amount of CENP-ACnp1 protein associated with chromatin is increased in the top3-105 mutant. Western blot of FLAG in wild type and the top3-105 mutant for untagged control strains and strains expressing CENP-ACnp1-FLAG from the endogenous locus. After lysis of cells histones were prepared by acid extraction from the insoluble chromatin faction. The soluble fraction was also included in the experiment. Western blot of histone H3 and actin were used as loading controls. (TIF) [file pgen.1003371.s004.tif]
