# Supplementary material for: DNA Topoisomerase III Localizes to Centromeres and Affects Centromeric CENP-A Levels in Fission Yeast
Source: PLoS Genet. 2013 Mar 14;9(3):e1003371. doi: 10.1371/journal.pgen.1003371 (PMC3597498; doi:10.1371/journal.pgen.1003371)
Supplement: Table S1 — Comparisons of chromosome segregation defects between strains. Lists of p values for pair wise comparisons of chromosome segregation defects between the indicated strains. The p values were generated using a two-tailed Fishers exact test. P values indicating a statistical difference between two strains are marked in bold numbers. (DOCX) [file pgen.1003371.s005.docx]

|  | **Total mitotic defects** | **Late anaphase**  **(lagging)** | **Telophase (unequal or cut/torn)** |
| --- | --- | --- | --- |
| ***top3-105***  **vs. WT** | **2,5E-11** | **1,9E-04** | **2,3E-07** |
| ***rqh1Δ***  **vs. WT** | **5,9E-06** | **1,0E-03** | **1,2E-02** |
| ***top3Δ rqh1Δ***  **vs. WT** | **1,4E-03** | 1,2E-01 | **2,6E-02** |
| ***rhp51Δ***  **vs. WT** | **5,9E-06** | **1,0E-03** | **1,2E-02** |
| ***top3-105 rhp51Δ***  **vs. WT** | **6,6E-05** | **5,3E-03** | **2,6E-02** |
| ***rqh1Δ rhp51Δ***  **vs. WT** | **2,6E-06** | **1,2E-02** | **4,4E-04** |
| ***top3-105***  **vs. *rqh1Δ*** | **2,4E-02** | 8,0E-01 | **8,0E-03** |
| ***top3Δ rqh1Δ***  **vs. *top3-105*** | **2,4E-04** | **4,8E-02** | **3,3E-03** |
| ***top3Δ rqh1Δ***  **vs. *rqh1Δ*** | 2,0E-01 | 1,4E-01 | 1 |
| ***top3-105 rhp51Δ***  **vs. *top3-105*** | **4,6E-03** | 4,4E-01 | **3,3E-03** |
| ***top3-105 rhp51Δ***  **vs. *rhp51Δ*** | 6,9E-01 | 7,9E-01 | 1 |
| ***rqh1Δ rhp51Δ***  **vs.  *rqh1Δ*** | 1 | 5,9E-01 | 4,2E-01 |
| ***rqh1Δ rhp51Δ***  **vs. *rhp51Δ*** | 1 | 5,9E-01 | 4,2E-01 |
