# Supplementary material for: DNA Topoisomerase III Localizes to Centromeres and Affects Centromeric CENP-A Levels in Fission Yeast
Source: PLoS Genet. 2013 Mar 14;9(3):e1003371. doi: 10.1371/journal.pgen.1003371 (PMC3597498; doi:10.1371/journal.pgen.1003371)
Supplement: Table S2 — Top3 has no major effects on transcription of genes known to affect nucleosome or CENP-ACnp1 dynamics. List of genes that display >1.5-fold up- or down-regulation of RNA levels in top3-105 compared to WT in the Gene Ontology categories GO:0007059 Chromosome Segregation, GO:0051276 Chromosome organization or GO:0000775 Chromosome, centromeric region (n = 460 genes). RNA levels in wild type and the top3-105 mutant and the ratio between these are shown. (DOC) [file pgen.1003371.s006.doc]

| **Systematic name** | **Name** | **Description** | **WT RNA (AU)** | ***top3-105* RNA (AU)** | **Ratio** |
| --- | --- | --- | --- | --- | --- |
| SPCC417.06c | mug27 | meiosis specific protein kinase Mug27/Slk1 | 107,85 | 385,33 | 3,57 |
| SPBC1711.14 | rec15 | meiotic recombination protein Rec15 | 61.90 | 185,80 | 3,00 |
| SPAC14C4.08 | mug5 | meiotically upregulated gene Mug5 | 125,32 | 376,15 | 3,00 |
| SPAC14C4.03 | mek1 | Cds1/Rad53/Chk2 family protein kinase Mek1 | 67,10 | 125,59 | 1,87 |
| SPAC15A10.10 | mde1 | Muskelin homolog (predicted) | 475,10 | 876,44 | 1,84 |
| SPAC1002.06c | bqt2 | bouquet formation protein Bqt2 | 57,83 | 105,30 | 1,82 |
| SPBC216.02 | mcp5 | cortical anchoring factor for dynein Mcp5/Num1 | 72,71 | 120,34 | 1,66 |
| SPAC1952.15c | rec24 | meiotic recombination protein Rec24 | 225,94 | 357,79 | 1,58 |
| SPBC1718.02 | hop1 | linear element associated protein Hop1 | 116,46 | 179,82 | 1,54 |
| SPBC16A3.19 | eaf7 | histone acetyltransferase complex subunit Eaf7 | 859,15 | 558,80 | 0,65 |
| SPCC297.04c | set7 | histone lysine methyltransferase Set7 (predicted) | 827,86 | 525,17 | 0,63 |
| SPAC19D5.11c | ctf8 | DNA replication factor C complex subunit Ctf8 (predicted) | 1627,44 | 1060,56 | 0,65 |
| SPCC622.19 | jmj4 | Jmj4 protein | 2241,02 | 1422,32 | 0,63 |
| SPCC645.11c | mug117 | meiotically upregulated gene Mug117 | 734,15 | 452,79 | 0,62 |
| SPAC14C4.12c | laf1 | clr6 L associated factor 1 Laf1 | 2378,64 | 1508,37 | 0,63 |
| SPBC2D10.13 | est1 | telomerase regulator Est1 | 1010,76 | 650,19 | 0,64 |
