# Supplementary material for: DNA Topoisomerase III Localizes to Centromeres and Affects Centromeric CENP-A Levels in Fission Yeast
Source: PLoS Genet. 2013 Mar 14;9(3):e1003371. doi: 10.1371/journal.pgen.1003371 (PMC3597498; doi:10.1371/journal.pgen.1003371)
Supplement: Table S3 — List of strains used in this study. Strain names and genotypes for all strains used in this study. The right column indicates in which figures each strain has been used. (DOC) [file pgen.1003371.s007.doc]

| **Strain** | **Genotype** | **Figure** |
| --- | --- | --- |
| HU0303 | *h-* | 1B, 1C, 2, 4, 6A-D, 6F S1, S2, S4 |
| HU2027 | *h+ top3-105ts ade6-M210, leu1-32, his2-* | 1B, 1C, 2, 4, 6A-D, S1, S2, S4 |
| HU2075 | *h- rqh1Δ:ura4+ leu1-32, ura4-D18* | 1B, 1C, 2, 6D |
| HU2306 | *h- top3Δ::ura4+ rqh1Δ::Kan leu1- ura4- his7-* | 1B, 1C, 2, 6D |
| HU2183 | *h- scm3-Pk::hph ade6-M210, leu1-32, ura4-D18* | 6E |
| HU2190 | *h- top3-105ts scm3-Pk::hph ade6-M210, leu1-32, ura4-D18* | 6E |
| HU2195 | *h- rqh1Δ:kan scm3-Pk::hph ade6-M210, leu1-32, ura4-D18* | 6E |
| HU2469 | *h+ top3Δ::ura4+ rqh1Δ::Kan Scm3-Pk::hph leu1-, ura4-, his7-* | 6E |
| HU1747 | *h- top3-myc::Kan ade6-M210, leu1-32, ura4-D18* | 3, 5, S2, S3 |
| HU2305 | *h- rhp51Δ::hph leu1-32, ura4-D18* | 6F |
| HU2325 | *h+ rhp51Δ::hph top3-105ts ade6-M210, ura4-D18, his2-* | 6F |
| HU2334 | *h- rhp51 Δ::hph rqh1Δ::ura4+ leu2-31, ura4-, his2-* | 6F |
| HU2282 | *h- cnp1-FLAG::Kan* | S4 |
| HU2466 | *h- top3-105ts cnp1-FLAG::Kan ade6-M210* | S4 |
