# Supplementary material for: DNA Topoisomerase III Localizes to Centromeres and Affects Centromeric CENP-A Levels in Fission Yeast
Source: PLoS Genet. 2013 Mar 14;9(3):e1003371. doi: 10.1371/journal.pgen.1003371 (PMC3597498; doi:10.1371/journal.pgen.1003371)
Supplement: Table S4 — List of primers used in this study. Sequencing and qPCR primers used in this study. The right column indicates which experiment each strain has been used in. (DOC) [file pgen.1003371.s008.doc]

**Table S4. List of primers used in this study.**

| **Primer** | **Sequence** | **Experiment** |
| --- | --- | --- |
| Top3_F1 | TGCCTTCTAGACAAGGTCGCT | Amplification and sequencing of *top3-105* |
| Top3_R1 | TCGACTTGCTTAACGGATCCAACT | Amplification and sequencing of *top3-105* |
| Top3_F2 | TCTGGTCCTCGATTTACTACCTT | Sequencing of *top3-105* |
| Top3_F3 | TCCCTCAAGACGTTTTATTCGATGC | Sequencing of *top3-105* |
| Top3_F4 | GATCGGTGGCAGCGAGTAGA | Sequencing of *top3-105* |
| Top3_F5 | GAGATTATCGACCGCCCCGA | Sequencing of *top3-105* |
| Top3_F6 | TCAGCGCTTCCTTCCCAAGA | Sequencing of *top3-105* |
| Top3_F7 | CCGAAACGTTGGTGCAGGTT | Sequencing of *top3-105* |
| Cnt1_1F | AGACAATCGCATGGTACTATC | ChIP-qPCR |
| Cnt1_1R | AGGTGAAGCGTAAGTGAGTG | ChIP-qPCR |
| Act1_F | CCCAAATCCAACCGTGAGAAGATG | ChIP-qPCR |
| Act1_R | CCAGAGTCCAAGACGATACCAGTG | ChIP-qPCR |

Sequencing and qPCR primers used in this study. The right column indicates which experiment each strain has been used in.
